# Surviving families: experiences of losing a family member to suicide*

**DOI:** 10.1590/1518-8345.7676.4614

**Published:** 2025-10-27

**Authors:** Elena Bustos Alfaro, Mara Regina Santos da Silva, Kateline Simone Gomes Fonseca, Carl Lacharité, Elga Mirta Furtado Barreto de Carvalho, Ariana Sofia Barradas da Silva

**Affiliations:** 1 Universidad de Costa Rica, Escuela de Enfermería, San Pedro, SJ, Costa Rica; 2 Universidade Federal do Rio Grande, Rio Grande, RS, Brazil; 3 Scholarship holder at the Coordenação de Aperfeiçoamento de Pessoal de Nível Superior (CAPES), Brazil; 4 Université Du Québec à Trois Rivières, Quebec, QC, Canada; 5 Universidade de Cabo Verde, Enfermagem, Praia, Cape Verde

**Keywords:** Family, Suicide, Completed, Resilience, Psychological, Primary Health Care, Family Nursing, Family Health

## Abstract

to identify the interactional processes that allow surviving families to cope with the death by suicide of one of their members, to restructure themselves as a unit and transform the experience into a learning experience.

a qualitative, exploratory study, guided by the concept of Family Resilience, in which eight families, who experienced the suicide of one of their members participated. Data were collected through semi-structured interviews and the data were submitted to thematic analysis.

the results were organized into four thematic groups and revealed grief, despair, and perplexity of family members who could not understand the reasons for such a radical act; the feelings of pain due to the loss, overlapped with anger, relief and guilt. Blame was shared with other people and the social and health services; and the lessons that the experience provides.

nurses and other healthcare professionals can help surviving families to restructure themselves after the suicide of one of their members, implementing care actions based on repeated assessments of the repercussions of suicide on the family as a whole and on its members individually; identifying the most compromised dimensions of family life; individual and family needs; and family resilience processes.

## Highlights

**(1)** The experience of suicide triggers a range of overlapping feelings within the family. **(2)** Lessons learned help the family (re)create ways of relating to the loss. **(3)** Health professionals must define care strategies based on ongoing assessments.

## Introduction

Suicide is one of the leading causes of death, affecting thousands of people worldwide. Due to its unexpected and violent nature, it is an experience marked by stigma^(1)^. Each family perceives and experiences it differently when a member dies by suicide. Some families face unbearable pain, while others, in addition to the pain, see it as the end of a cycle of hardship and the possibility of easing or resolving long-standing problems.

It is estimated that at least five to six people close to someone—usually family members—are directly affected when a person dies by suicide, and it is often an act they cannot fully understand^(2-3)^. Considering that approximately 700,000 suicides occur each year worldwide, around four million people are expected to go through this experience, representing a large contingent of exposed families who may react to the phenomenon in different ways^(1)^.

The devastating nature of this experience is exacerbated by the fact that these families are generally not the focus of care within the health system, making them a potentially vulnerable population for developing physical and mental health problems, including suicidal thoughts or the risk of dying by suicide themselves^(4-5)^. Attention is often centered on the person who died, and after the tragic outcome, family members are left without support from health services—precisely when they are experiencing their greatest vulnerability, marked by intensified feelings of guilt and anger^(6-7)^.

For this reason, nurses and other healthcare professionals, especially those working in the Family Health Strategy (FHS), should assist these families and support them in coping. They should identify how each family responds to the loss, assess their needs, and monitor the repercussions that suicide may have on surviving family members. Therefore, the FHS plays a crucial role in these families’ grieving and recovery processes, precisely because it operates on a territorial and community basis, with facilitated access that enables FHS professionals to understand the individual needs of each family^(4)^.

A review of national publications on the subject revealed an increase in suicide rates across all Brazilian regions, reaching 7.27 per 100,000 inhabitants in 2022, with the South presenting the highest rate: 11.53 per 100,000 inhabitants^(8-9)^. Studies highlight the impact of suicide on bereaved survivors, including the emergence or worsening of conflicts that may lead to marital and emotional breakdowns; compromised financial resources, especially when the deceased was the family’s provider; as well as the development of disorders such as anxiety, depression, and the abuse of legal and illegal substances. At the same time, there are few guidelines to inform healthcare professionals’ practices^(8-10)^, particularly regarding care actions and strategies, revealing a significant gap in postvention efforts^(4)^.

Although international studies provide detailed accounts of families’ needs and available support resources, particularly in developed countries such as the United States, Australia, and Canada, where consolidated policies and programs are in place, authors often report that actions are generally short-term. They emphasize that care must be improved to ensure it is effective, sensitive, flexible, and sustained over the long term^(5,11-12)^.

Based on the previous discussion, this study focuses on the processes that support families in restructuring after the suicide of one of their members, considering that this restructuring depends on the type of support provided from the moment the suicide is discovered until the family is able to recover. Some families may lack the necessary resources to effectively cope with such an adverse situation. In others, however, the suicide of a family member may trigger not only a mourning process but also the reorganization and restructuring of the family unit, enabling its members to build emotional strength for managing future adversities^(12-13)^.

This situation relates to the concept of family resilience, which helps explain how families face adversities that affect their functioning, such as the loss of a family member by suicide, and restructure themselves as a functional unit, supported by interactive processes of communication, organization, and their own belief system^(13)^. Organizational processes involve, among other elements, flexibility to adapt to new situations and connection among members; communication processes involve open emotional expression, clarity, and cooperation in problem-solving; and the belief system refers to the family’s cultural and religious framework, which enables them to find meaning in adversity through a perspective of trust and hope^(13)^. This concept is essential for understanding how families (re)organize themselves based on adverse experiences and transform them into learning. In this sense, it has the potential to inform interventions aimed at helping families restructure after a traumatic event.

The objective of this study is to identify the interactional processes that allow surviving families to cope with the death of a member by suicide, restructure themselves as a unit, and transform the experience into learning.

## Method

### Study design

This qualitative and exploratory study adopted Froma Walsh’s concept of Family Resilience^(13)^. This concept is applicable to studies involving families facing adverse situations that significantly impact their functioning but who, despite the suffering they endure, are able to restructure themselves as a functional unit and accumulate experiences to help face challenges in later stages of their life cycle.

### Study setting

The study was conducted in Rio Grande, RS, Brazil—a city with high suicide rates according to the Notification System, reaching 11.55 per 100,000 inhabitants. The most frequently reported causes include poor economic conditions, illicit drug use, and depressive or anxiety disorders^(10,14)^.

### Period

Data were collected between May and August 2022.

### Study population

Eight families, represented by one or two of their members, participated in the study. A search of death certificates (DCs) related to suicide was conducted to locate these families. Initially, DCs recorded in the city’s Mortality Information System of the Epidemiological Surveillance Service between January 2016 and December 2021 (the period for which data were available) were reviewed. The search identified 123 families.

Data collected from the DCs—such as the deceased’s name, date of birth, and parents’ names—made it possible to locate and contact close relatives through social media platforms (Facebook and Instagram). In this stage, 14 families were contacted, but only three agreed to participate. Another six family members were identified through the Management System of the Municipal Health Department of Rio Grande, RS (G-MUS). Although they accepted the invitation, only three attended the interviews. In a third attempt to expand the sample, the G-MUS was accessed again, and another 11 family members were located; only two agreed to participate in the study. Families who declined to participate explained that too much time had passed since the suicide and they no longer wished to relive the painful emotions associated with the loss.

The sample comprised information from eight families, intentionally selected based on the following criteria: having experienced the suicide of a family member within the five years prior to the start of data collection; residing in the city where the study was conducted; and being 18 years of age or older to participate in the interview. The exclusion criterion was the presence of an emotional condition that could compromise the participant’s ability to engage in the interview or discuss the subject. The eight families were identified using a code consisting of the letter “F” followed by a number corresponding to the order in which the interviews were conducted.

### Data collection

Data were collected through semi-structured interviews conducted privately and in person with seven families, and online with one. All interviews were audio recorded and conducted by the first and third authors of this article—registered nurses, doctoral students, and residents of the region where the study was carried out. They are members of a research group that works with families in vulnerable situations. Both had experience in collecting qualitative data and no prior contact with the participants.

A four-part script guided the interviews. The first part was designed to characterize the informants, addressing age, sex, education, and their relationship with the person who died by suicide. The second part characterized the family in terms of income and main provider. The third part explored how the respondent and other family members experienced the loss; what suicide represented—or still represents—in the family’s life; what helped the family restructure; the changes that occurred in the family’s daily life after the suicide; and how the family is currently organized. The fourth part investigated where and/or from whom family members sought help before, during, and after the suicide; the family’s strengths; and the lessons learned from the experience. This script was previously tested, and no changes were necessary.

The interviews lasted an average of 2 hours and 40 minutes. Two meetings were held with four families and three meetings with the others, totaling 21 hours and 50 minutes of data collection. At each new meeting, participants were invited to validate the summarized information. All were invited to a public presentation of the results, and one participant (F2) attended the event.

### Data analysis

Data were subjected to thematic analysis^(15)^, following these steps: pre-analysis, material exploration, and result processing. Initially, repeated readings of all interview material were conducted to identify significant patterns and expressions linking the experience of suicide to elements of family resilience processes, such as cohesion and flexibility. In the second stage, the goal was to interpret the meanings embedded in the previously identified expressions and create groupings. The material was then reorganized into four thematic cores, using as criteria a temporal dimension and the meaning attributed by the participant to the loss of their family member by suicide. Five researchers participated in the analysis process at different stages, through face-to-face and online discussions, with no disagreements.

## Ethical aspects

The study was approved by the Institutional Review Board at the Federal University of Rio Grande (Opinion: 5,037,888) and by the Municipal Center for Public Health Education of the city’s Health Department, under Opinion 13/2022. The ethical principles outlined in Resolution 510/2016 of the Brazilian National Health Council were followed, and all participants signed a free and informed consent form.

## Results

Seven of the families who participated in this study were represented by one member, and one family (F6) had two representatives: the mother and the stepfather. Among the individuals who died by suicide, only one was female (F8). The majority chose hanging as the method (F2, F3, F4, F5, F6, F7, and F8), while only F1 died by drug intoxication. The individuals from F2 and F6 had been diagnosed with schizophrenia; F4 and F8 with depression; and F1 with epilepsy. Five had used illicit drugs, including cocaine and crack (F1, F2, F4, F5, F7, and F8), and marijuana (F6); three also abused alcohol (F4, F6, and F7).

The time elapsed between the suicide and data collection ranged from seven months (F4 and F6) to four years (F8); one year (F3); two years (F2 and F7); and three and a half years (F1 and F5). Additional participant characteristics are presented in Figure 1.

**Figure 1–:** Participants’ sociodemographic characteristics (n* = 9). Rio Grande, RS, Brazil, 2022

**Sociodemographic characteristics**								
**Code**	**Relationship with the deceased**	**Age (years)**	**Sex**	**Marital status**	**Ethnicity**	**Religion**	**Occupation**	**Education**
**F1**	Mother	40	F ^†^	Married	Black	Jehovah’s Witness	Housekeeper	High school
**F2**	Sister	30	F ^†^	Married	White	Spiritism	Homemaker	Middle school
**F3**	Cousin	24	M ^‡^	Single	White	Catholic	Health community agent	Bachelor’s degree
**F4**	Wife	38	F ^†^	Widowed	Black	Evangelical	Confectioner	High school
**F5**	Sister	27	F ^†^	Stable union	Black	*Umbanda*	Unemployed	Middle school
**F6-1**	Mother	66	F ^†^	Stable union	Black	Catholic	Retired, family caregiver	High school
**F6-2**	Stepfather	58	M ^‡^	Stable union	Black	Catholic	Retired	Primary school
**F7**	Wife	39	F ^†^	Widowed	Mixed race	*Macumbeira* ^§^	App driver	High school
**F8**	Wife	44	F ^†^	Widowed	White	Spiritism	Cook	High school

*n = Participants; ^†^F = Female; ^‡^M = Male; ^§^Although the term may carry a pejorative connotation, it was used by the participant to indicate she is a practitioner of an Afro-Brazilian religion

The results of this study are presented in four thematic groups: (1) *Suicide as an act that impacts and goes beyond comprehension;* (2) *Shared guilt;* (3) *Overlapping feelings regarding death by suicide; and* (4) *Lessons learned from the experience.* The first group portrays the impact that the confirmation of the suicide had on individual family members and the family as a whole. The second and third groups explore the emotions experienced by family members, including grief over the loss, interwoven with anger, relief, and guilt— the latter imputed to others as well, including social and health services. The fourth group focuses on the lessons that emerged from the experience.

The first thematic core, *Suicide as an act that impacts and goes beyond comprehension*, reveals reactions such as grief, despair, and bewilderment experienced by family members when confronted with the suicide. All eight families described the experience as tragic and reported being unable to understand the reasons that led their loved one to make such an extreme decision.

Although suicide was a possibility present in the daily lives of some families, they described the moment of realization as unexpected, as they believed they had taken preventive measures. The mother in F1 had removed objects such as ropes that could be used for hanging from her son’s reach; however, he died by drug intoxication. The sister in F2 reported that she had admitted her brother to a psychiatric hospital, believing he would be safe there. The mother in F6 monitored changes in her son’s routine—he lived in the same courtyard—but only discovered the suicide two days after it had occurred.

The following statements illustrate the impact of discovering the suicide: *Because it was suicide, I think it was a shock, because no one is prepared to experience an event like that in the family* (F3); *I opened the door and there he was: his body hanging. Imagine that! I was in despair* (F4); *I thought: ‘he has nowhere to put a rope, I don’t have anything like that at home, his room doesn’t have anything either’... But he had already planned what to do. He took all of his stock of Gardenal* [phenobarbital] *that we had at home* (F1); *Every morning before going to work, I would go to his door and take a look, I would see the light on, the door open and I thought everything was fine... When I saw the door closed, I was afraid he was going to do something. Two days went by without me seeing him... but he was already hanging there* (F6).

The second thematic group, Shared guilt, portrays the feeling of guilt experienced in the aftermath of a loved one’s suicide. This feeling is expressed by family members in a way that distributes responsibility across different contributors to the outcome, resembling a process of shared attribution. It involves three perspectives: guilt felt by the participant; guilt attributed to others who were part of the deceased’s life; and guilt directed toward social and health services from which support had been sought.

Participants reported feeling that they had, in some way, contributed to their family member’s suicide—whether by failing to recognize signs that the person was unwell, not taking concrete actions to prevent the death, or not becoming more involved in the person’s life and thus providing more effective support.

The second perspective attributes blame to other people who, in the participant’s view, contributed significantly to the family member’s decision to end their life. This situation is generally marked by physical and emotional distancing from significant others, such as parents and partners, either due to the prioritization of work or a deliberate choice to limit contact in order to avoid conflicts—mainly related to drug use and violence.

The third perspective involves the social and health services to which the person who committed suicide was connected. The eight people who committed suicide were addicted to illicit drugs or had been diagnosed with a mental disorder and, in some way, were connected to the local Psychosocial Care Center for Alcohol and Other Drugs (CAPS AD) or to public safety services. However, the slowness of care in these services meant that the family only managed to get a referral for hospitalization after the suicide was completed. *I filed [the police report] but it didn’t work! So much so that only now is paperwork for his hospitalization arriving... Now they want a hearing! Are they going to hold a hearing without him here? I think that if they had done something, he would still be here today* (F5). *He [the person who committed suicide] spent the whole night going to his brother’s house asking for money and the last time he went to ask, he refused to give him the money. Less than half an hour later we called to say that he was dead. The brother felt guilty for not having given it and he carries that with him to this day* (F5).

All eight families reported experiencing guilt, with its intensity varying over time. This variation was influenced by the history previously shared with the deceased, how each family member processed the pain, and their individual beliefs and values.

The third thematic group, *Overlapping feelings in the face of death by suicide*, refers to the pain of loss, compounded by other emotions such as a sense of failure in the caregiving and protective role—especially when the family bond involved a parent-child or sibling relationship. It also encompasses anger and a desire for revenge, directed at individuals perceived to have contributed to the tragic outcome, such as in relationships involving drug users and drug dealers. Additionally, some participants expressed a sense of relief, as the death marked the end of a cycle of violent or destructive relationships. These emotions coexist with the grief over the loss and are shaped by the nature of the bond with the deceased, the shared history, and the time elapsed since the suicide, as illustrated by the following excerpts: *He [the father] understands that he failed, but it is as he says: he was disconnected from reality! But he understands that he failed and that he did not talk, because if you do not talk to your son at home, he will look for people on the street to talk to* (F1); *In my case, it was both painful and a relief. Not because I got rid of the person, but because there were threats and people after him* (F7); *One day that was good for me was when they killed the drug dealer who was luring my son. That was the best day! Because I’m going to cry, but his mother is going to cry too. We shouldn’t wish this on others, but it’s a moment of revolt* (F1).

Participants from families F1, F2, F4, F5, F6-1, F6-2 and F8 report that the first moments after the event are extremely painful, as one mother described: *It seems the pain won’t go away! You carry it with you all the time. You wake up in the morning and think of the person and miss them* (F1).

The fourth thematic group, *Lessons from experience*, shows that suicide becomes a turning point integrated into the family’s collective experience, with the potential to bring about changes in the dynamics and behavior of its members. Among the lessons mentioned by participants are the need to prioritize family life; make adjustments to daily routines to ease pain and longing; learn to manage personal feelings of pain, guilt, and anger; and preserve and value the positive aspects of the relationship with the person who died.

These teachings play a relevant role in the family restructuring process, with survivors feeling more competent to prevent or reduce the likelihood of suicide recurrence among other family members. They also help the family (re)create a new way of relating to the loss and absence of the person who died, as the following statements indicate: *You learn to improve from death (F1); Pain does not last forever... we learn to live and hold on to everything that was good, because what was good comforts and helps us move forward* (F2); *I managed to stop blaming myself and asking myself: Where did I fail? What did I do? Why was I selfish? I decided at that moment to put an end to it. There are so many questions that have no answers! There is no going back! But I want to live, so I will not nullify myself* (F7); *I am here today* [*in the interview] because I would like to understand things better to help my daughter. You know! She tried to commit suicide five times* (F8).

## Discussion

The suicide of a loved one—such as a child, parent, sibling, or partner—elicits a range of reactions and feelings of pain and despair within the family, along with concurrent emotions such as guilt, anger, and relief, as reflected in the four thematic groups presented in this study. These are difficult emotions to endure, and they may persist for varying lengths of time for each individual, until a point of restructuring is reached.

Not all families who experience a loss by suicide are able to restructure themselves as a functional unit, but those that do exhibit certain characteristics of family resilience, including the presence of an adverse context marked by pain, suffering, and despair^(13)^, as shown in the first theme. When the suicide was confirmed, families reported feelings of despair, accompanied by questions about why the person ended their life. However, the findings of this study indicate that, over time, these feelings take on new contours, and the family begins to recreate ways of relating to the loss and absence.

A study conducted in Sweden with surviving families corroborates these findings, showing that even though families were aware of their loved one’s mental health condition and expressed desire to die, they believed the person would not actually commit suicide and reported feeling shocked and struggling to carry out the tasks required by the situation^(16)^.

Notably, when suicide occurs in one’s own home, it is often the family members who first find the body, and in such cases, the violence of this revelation adds an additional emotional burden to the survivors^(17-18)^, as reported by seven families in our study who experienced the same situation.

Another study on the perceptions of children under six years of age regarding their parents’ suicide showed that they felt confused—not necessarily about the suicide itself, but about the emotional reactions of the adults around them. Many reported difficulty understanding what had actually happened^(19)^.

The confirmation of a suicide is undoubtedly a critical moment, influenced by the circumstances surrounding the death, and demands greater attention from the entire family, including members of all ages. In such situations, it is important to recognize that interactions among family members, friends, and health services can provide essential support, reinforcing the understanding that resilience is shaped by a network of relationships and shared experiences.

Regarding the feelings of guilt generated by the suicide of a family member, it is important to emphasize that, although such feelings may be shared, they are not mutually exclusive. Feeling guilty—whether due to a belief that one did not fulfill the caregiving role adequately—does not preclude assigning blame to health services for perceived negligence or acknowledging the influence of third parties. It is difficult for families not to feel guilty about the death of a loved one^(20-21)^, even when suicide was known to be a possibility. Parents, in particular, often feel responsible for their child’s decision to end their life^(22)^, and in such cases, sharing the blame may, to some extent, alleviate the sense of shame and perceived failure in fulfilling a protective role. However, sharing blame does not eliminate the feeling of guilt.

Although sharing guilt can bring some relief, authors recommend caution, as guilt should not persist to the point of compromising the family’s ability to resume daily routines^(23)^. Sharing feelings in support groups with others who have gone through similar experiences can help families better understand the factors that contributed to the suicide and support the coping and reorganization process. In this context, support groups for family members serve as spaces where they learn to manage self-blame, recognize their own needs, and discover strategies for coping with grief^(24)^. In summary, experiencing and sharing guilt, while processing personal emotions, can contribute to the family’s reorganization and promote changes in family functioning^(23)^.

The third thematic group shows that death by suicide creates space for the emergence of feelings beyond the pain of loss—feelings that are not always considered socially or culturally acceptable. One such feeling is the frustration of not having been a “good enough” father, capable of giving the child a reason to live. This sentiment, clearly expressed by participants F1 and F2, is supported by other studies that describe a sense of shame leading parents to withdraw socially, which can result in physical and mental health problems and, in some cases, suicide attempts. The literature indicates that this behavior is more pronounced among first-degree relatives and occurs more frequently in fathers than in mothers^(23,25)^.

Another feeling that overlaps with the pain of loss is anger—often directed at the person who died for abandoning those left behind; at oneself for not preventing the death^(20-21)^; or at God and other uncontrollable forces, due to the inability to make sense of the suicide^(26)^. Other studies have also reported anger directed at health professionals who cared for the deceased^(27)^. In the present study, anger was also associated with individuals involved in drug use and trafficking, within a context where debt collection and threats against the family played a decisive role in the person’s decision to end their life. In these cases, alongside the feeling of anger, participants expressed a desire for revenge and an added sense of guilt for not having prevented their child from following the path of addiction.

The feeling of relief, superimposed on the pain of loss, emerged in families where the relationship with the person who died by suicide was abusive. In such cases, consistent with the literature, death was seen as a way to end a cycle of violence^(28)^. Similarly, relief was reported when death brought an end to the suffering of individuals with incapacitating or debilitating illnesses^(27)^, as well as in situations involving concern for the safety and well-being of a loved one, particularly in the context of drug use and trafficking. In these scenarios, over time, families manage to establish a “new normal” and even find motivation to support others facing similar challenges^(29)^.

The fourth thematic group reflects a more advanced stage in the coping process experienced by the families in this study. At this point, certain elements that support families in overcoming the pain of absence become more apparent, including assigning new meaning to the loss; reviewing and rebuilding intra-family relationships to strengthen cohesion among members; and redefining parenting approaches, particularly in cases where the kinship bond was between parents and children.

Authors report that the family reorganizes itself by often reassessing the roles of its members and parental responsibilities^(29-30)^, which contributes to the development of experience and skills to better care for oneself and others. They also emphasize that preserving positive memories of the relationship with the person who died helps to reframe the experience and uphold the dignity of the loved one^(12)^. Over time, the need to search for justifications for the suicide tends to diminish, making room for new forms of family interaction^(30-31)^.

As a contribution of this study, it is worth highlighting that focusing on the interactional processes that support the restructuring of families who have experienced the loss of a member by suicide shifts the perspective from viewing them as problematic or unstructured to recognizing their potential to cope, grow stronger, and learn from the experience. It also serves as a reminder that the death of the person “registered in the service” does not mark the end of the responsibility to care for the grieving family. This is particularly relevant given the high rates of suicide and suicide attempts among individuals with a family history of previous attempts. In this context, providing care to the grieving family is a preventive action.

Regarding limitations, it is important to note that this study involved a small sample and included only representatives of bereaved families in the interviews, which limited the identification of the interactional processes mobilized by other segments of the family.

## Conclusion

The loss of a family member by suicide is an experience that can compromise the family unit if its members do not receive support to address the problems that the situation brings to light. This study found that intervention with surviving families can reduce the time needed for their restructuring and that Primary Health Care is the setting in which care actions can be effectively implemented, given the ease with which teams can identify and access these families.

In this context, nurses and other health professionals must define care actions based on repeated assessments of the impact of suicide on the family as a whole and on each member individually; on identifying the most compromised aspects of family life; on both individual and collective needs; and on mobilizing interactional processes of communication and organization. These processes support, among other things, the sharing of pain and emotions—often contradictory feelings of guilt, anger, and failure—either among family members or within support groups. They also contribute to the reorganization of the family as a whole, allowing for the reconfiguration of each member’s place and role. It is important to emphasize that caring for the surviving family does not eliminate the need for individualized support, as each person responds differently depending on the bond and the history shared with the deceased.

## Data Availability

All data generated or analysed during this study are included in this published article.
