# Supplementary material for: Surviving families: experiences of losing a family member to suicide
Source: Rev Lat Am Enfermagem. 2025 Oct 27;33:e4614. doi: 10.1590/1518-8345.7676.4614 (PMC12562581; doi:10.1590/1518-8345.7676.4614)
Supplement: Translation

# Translation: Famílias sobreviventes: a experiência de perder um de seus membros por suicídio*

**DOI:** 10.1590/1518-8345.7676.4615

**Authors:** Elena Bustos Alfaro, Mara Regina Santos da Silva, Kateline Simone Gomes Fonseca, Carl Lacharité, Elga Mirta Furtado Barreto de Carvalho, Ariana Sofia Barradas da Silva

**Affiliations:** 1 Universidad de Costa Rica, Escuela de Enfermería, San Pedro, SJ, Costa Rica; 2 Universidade Federal do Rio Grande, Rio Grande, RS, Brasil; 3 Bolsista da Coordenação de Aperfeiçoamento de Pessoal de Nível Superior (CAPES), Brasil; 4 Université Du Québec à Trois Rivières, Quebec, QC, Canadá; 5 Universidade de Cabo Verde, Enfermagem, Praia, Cabo Verde

**Keywords:** Família, Suicídio Consumado, Resiliência Psicológica, Atenção Primária à Saúde, Enfermagem Familiar, Saúde da Família

## Abstract

identificar os processos interacionais que permitem às famílias sobreviventes enfrentarem a morte por suicídio de um de seus membros, se reestruturarem como unidade e transformarem a experiência em aprendizado.

estudo qualitativo, exploratório, orientado pelo conceito de Resiliência Familiar, no qual participaram oito famílias, que vivenciaram o suicídio de um de seus membros. A coleta de dados foi conduzida por meio de entrevistas semiestruturadas e os dados foram submetidos à análise temática.

apresentados em quatro núcleos temáticos, mostram as reações de pesar, desespero e perplexidade dos familiares que não conseguem entender as razões para um ato tão radical; os sentimentos de dor pela perda, sobrepostos com raiva, alívio e culpa, este último partilhado com outras pessoas e os serviços sociais e de saúde; e os ensinamentos que a experiência propicia

os enfermeiros e demais profissionais da saúde podem auxiliar as famílias sobreviventes a se reestruturarem após o suicídio de um de seus membros com ações de cuidado planejadas a partir de avaliações repetidas da repercussão do suicídio na família na totalidade e sobre seus membros individualmente; da identificação das dimensões da vida familiar mais comprometidas; das necessidades individuais e familiares; e dos processos de resiliência familiar.

## Destaques

**(1)** A experiência do suic**í**dio na família desencadeia uma gama de sentimentos sobrepostos. **(2)** Os ensinamentos ajudam a família (re)criar uma forma de se relacionar com a perda. **(3)** Profissionais da saúde devem definir o cuidado a partir de avaliações constantes.

## Introdução

O suicídio é uma das principais causas de morte ao redor do mundo, atingindo milhares de pessoas e constituindo-se em uma experiência marcada por estigmas devido à sua natureza inesperada e violenta^(1)^. Quando cometido por um dos membros da família, apresenta-se como uma experiência única, uma vez que cada família a percebe e sente de forma diferente. Para algumas famílias, representa uma dor imensurável, enquanto para outras pode significar, além da dor, o final de tempos difíceis e a perspectiva de eliminar ou amenizar problemas que vivenciavam por longo tempo.

Estima-se que pelo menos cinco a seis pessoas próximas, geralmente da família, são afetadas diretamente quando acontece o suicídio de uma pessoa, sendo este um ato que muitas vezes elas não conseguem entender^(2-3)^. Considerando que ocorrem aproximadamente 700.000 suicídios por ano no mundo, calcula-se que cerca de quatro milhões de pessoas estão vivenciando essa experiência, o que representa um grande contingente de famílias expostas que podem reagir de diversas formas ante este fenômeno^(1)^.

Contribui para exacerbar o caráter devastador dessa experiência o fato de essas famílias, em geral, não serem o foco dos cuidados no sistema de saúde, convertendo-se em uma população potencialmente vulnerável para desenvolver problemas de saúde física e mental, incluindo ideias ou risco de suicídio entre seus integrantes^(4-5)^. Muitas vezes, a atenção estava centrada na pessoa que cometeu o suicídio e, após o desfecho trágico, os familiares ficam desamparados pelos serviços de saúde, justamente por se encontrarem em um período de maior fragilidade, com sentimentos de culpa e raiva exacerbados^(6-7)^.

Por essa razão, é imprescindível que os enfermeiros e outros profissionais, especialmente da Estratégia Saúde da Família (ESF), se ocupem dessas famílias para ajudá-las no enfrentamento dessa experiência. Nesse sentido, é importante identificarem como cada uma reage diante de perda e, especialmente, as repercussões que o suicídio provoca na família sobrevivente, assim como as necessidades que apresentam. É nesta perspectiva que a ESF tem papel fundamental no processo de luto e restruturação dessas famílias, justamente porque atua em uma base territorial e comunitária que facilita o acesso e possibilita conhecer as necessidades de cada uma^(4)^.

Em uma busca nas publicações nacionais sobre o tema, constatamos aumento nas taxas de suicídio em todas as regiões brasileiras, atingindo 7,27 por 100 mil habitantes, em 2022, com a região sul apresentando o maior o índice: 11,53 por 100 mil habitantes^(8-9)^. No que tange à família, os estudos dão ênfase ao impacto do suicídio nos sobreviventes enlutados, incluindo o surgimento ou exacerbação de conflitos que podem levar a rupturas conjugais e afetivas; o comprometimento dos recursos financeiros, principalmente se o papel que a pessoa falecida ocupava na família era de provedor; além de transtornos como ansiedade, depressão, abuso de substâncias lícitas e ilícitas, entre outros. Constata-se, por outro lado, uma lacuna em termos de subsídios para orientar a prática dos profissionais^(8-10)^ com estas famílias enlutadas, notadamente em relação às ações e estratégias de cuidado, ou seja, é uma lacuna importante em termos de posvenção^(4)^.

Na literatura internacional, embora se encontre um maior detalhamento das necessidades das famílias e dos recursos de apoio disponíveis, principalmente em países desenvolvidos como Estados Unidos, Austrália e Canadá, onde existem políticas e programas consolidados, os autores referem que as ações são geralmente de curto prazo e que os cuidados precisam ser qualificados no sentido de torná-los resolutivos, sensíveis, flexíveis e de longo prazo^(5,11-12)^.

Com base nessas considerações, direcionamos este estudo para os processos que sustentam a reestruturação da unidade familiar subsequente ao suicídio de um de seus membros, sem perder de vista que esta reestruturação depende do tipo de suporte que as famílias encontram desde o momento em que constatam o suicídio até que consigam se recuperar. Algumas famílias podem não dispor dos recursos necessários para enfrentar esta situação adversa de forma efetiva. Em outras, entretanto, o suicídio de um de seus membros desencadeia não só um processo de luto, mas também de reorganização e reestruturação como unidade familiar, permitindo que seus integrantes acumulem uma bagagem de experiências para administrar outras adversidades posteriormente^(12-13)^.

Esta situação remete à concepção de resiliência familiar, a qual possibilita compreender a forma como a família enfrenta adversidades que impactam seu funcionamento, como a perda de um familiar por suicídio, e reestruturar-se como unidade funcional, apoiada em processos interacionais de comunicação, organização e seu próprio sistema de crença^(13)^. Os processos organizacionais envolvem, entre outros elementos, a flexibilidade para adaptar-se a novas situações e a conexão entre seus membros; enquanto que os processos de comunicação estão atrelados a expressão emocional aberta, com clareza e cooperação na resolução dos problemas; o sistema de crenças está vinculado ao arcabouço de crenças culturais e religiosas da família, que lhe permite extrair significados da adversidade, com uma perspectiva de confiança e esperança^(13)^. Trata-se de um conceito basal para melhor compreender como as famílias se (re)organizam a partir de experiências adversas que vivenciam e as transformam em aprendizado. Nesse sentido, tem potencial para subsidiar a intervenção com essas famílias, visando ajudá-las a se reestruturar após o evento traumático.

O objetivo deste estudo é identificar os processos interacionais que permitem às famílias sobreviventes enfrentarem a morte por suicídio de um de seus membros, se reestruturarem como unidade e transformarem a experiência em aprendizado.

## Método

### Tipo de estudo

Estudo qualitativo, do tipo exploratório, que utilizou como referência teórica o conceito de Resiliência Familiar de Froma Walsh^(13)^, uma vez que este se aplica a estudos com famílias que experienciam situações adversas, com forte impacto em seu funcionamento, mas apesar do sofrimento que experimentam conseguem se reestruturar como unidade funcional e agregar experiência para enfrentar desafios, em etapas posteriores do seu ciclo vital.

### Local do estudo

O estudo foi desenvolvido em Rio Grande-RS, Brasil, município onde, de acordo com o Sistema de Notificação, encontram-se altas taxas de suicídio, chegando a 11,55 por 100 mil habitantes, cujas causas mais frequentemente são a baixa condição econômica das famílias, o consumo de drogas ilícitas e os transtornos depressivos e de ansiedade^(10,14)^.

### Período

Os dados foram coletados no período entre maio e agosto de 2022.

### População do estudo

Participaram do estudo oito famílias, representadas por um ou dois de seus membros. Para localizá-las, inicialmente foi realizado um levantamento das Declarações de Óbito (DO) por suicídio, no Sistema de Informação sobre Mortalidade do Serviço de Vigilância Epidemiológica do município, registradas entre janeiro de 2016 e dezembro de 2021 (período em que os dados estavam disponibilizados para consulta). Este levantamento indicou que em 123 famílias havia ocorrido um caso de suicídio durante esse período.

Dados obtidos nos DO, como nome, data de nascimento do falecido e nome dos pais, possibilitaram localizar alguns parentes próximos e contactá-los através das redes sociais (*Facebook* e *Instagram*). Nesta etapa, foram contactadas 14 famílias, mas apenas três aceitaram participar do estudo. Posteriormente, foi utilizado o Sistema de Gestão da Secretaria Municipal de Saúde de Rio Grande-RS (G-MUS) e foram localizados seis familiares que aceitaram o convite, mas somente três compareceram às entrevistas. Em uma terceira tentativa de ampliar a amostra, o G-MUS foi novamente acessado e foram localizados 11 familiares. Destes, somente dois concordaram participar do estudo. As famílias que recusaram participar justificaram pelo fato de já ter se passado muito tempo desde o suicídio e não mais desejarem reviver os sentimentos dolorosos que a perda lhes trouxe.

A amostra ficou constituída pelas informações de oito famílias, selecionadas de forma intencional, tendo por base os critérios: ter experenciado o suicídio de um familiar nos cinco anos anteriores ao início da coleta de dados; residir no município onde o estudo foi realizado; ter 18 anos ou mais para participar da entrevista. Como critério de exclusão, foi considerado o familiar encontrar-se em uma condição emocional que comprometesse a interação para realização das entrevistas ou para falar sobre o assunto. As oito famílias foram identificadas com um código formado pela letra “F” seguida de um numeral correspondente à ordem de realização das entrevistas.

### Coleta de dados

Os dados foram coletados a partir de entrevistas semiestruturadas, realizadas privativamente, de forma presencial com sete famílias e com uma no formato *online*. Todas as entrevistas foram gravadas (em áudio) e conduzidas pela primeira e terceira autoras deste artigo, enfermeiras, doutorandas, residentes na região onde o estudo foi desenvolvido, integrantes de um grupo de pesquisa que trabalha com famílias em situação de vulnerabilidade e ambas com experiência prévia em coleta de dados qualitativos e sem contato prévio com os participantes.

Para guiar as entrevistas, utilizou-se um roteiro constituído de quatro partes. A primeira, destinada à obtenção de dados que caracterizam o informante, em relação a idade, sexo, escolaridade e parentesco com a pessoa que se suicidou. A segunda, voltada para a caracterização da família em termos de renda e principal provedor. A terceira parte abordava como o respondente e os demais membros da família vivenciaram a perda do familiar; o que o suicídio representou, ou ainda representa, na vida da família; o que ajudou a família a se reestruturar; as modificações que aconteceram no cotidiano da família após o suicídio; e como a família está organizada atualmente. A quarta parte investigou onde e/ou com quem os familiares procuraram ajuda antes, durante e após a pessoa ter se suicidado; os pontos fortes da família; e os aprendizados que extraíram da experiência. Este roteiro foi previamente testado, sendo que não houve necessidade de alterações.

As entrevistas duraram em média duas horas e quarenta minutos. Com quatro famílias foram realizados dois encontros e com as demais, três encontros, totalizando 21 horas e 50 minutos de coleta de dados. A cada novo encontro, os participantes eram convidados a validar as informações sintetizadas. Todos foram convidados para a apresentação pública dos resultados, sendo que uma participante (F2) compareceu na ocasião.

### Análises dos dados

Os dados foram submetidos à análise temática^(15)^, seguindo as etapas: pré-análise; exploração do material; e tratamento dos resultados. Inicialmente, foram realizadas leituras sucessivas de todo o material gerado nas entrevistas, procurando identificar padrões e expressões significativas, que associavam a experiência do suicídio e elementos dos processos de resiliência familiar como coesão, flexibilidade, entre outros. Na segunda etapa, buscou-se apreender os sentidos integrados nas expressões identificadas anteriormente e criar agrupamentos. Posteriormente, o material foi reagrupado em quatro núcleos temáticos, utilizando como critérios uma dimensão temporal e o sentido atribuído pelo participante à perda do familiar por suicídio. Cinco pesquisadores, em diferentes momentos, participaram do processo de análise que foi conduzido a partir de discussões presenciais e no formato *online*, sem discordâncias.

## Aspectos éticos

O estudo recebeu certificação do Comitê de Ética em Pesquisa com Seres Humanos da Universidade Federal do Rio Grande (Parecer: 5.037.888) e do Núcleo Municipal de Educação em Saúde Coletiva da Secretaria Municipal de Saúde, sob parecer 13/2022. Foram respeitados os preceitos éticos da Resolução n.^o^ 510/2016 do Conselho Nacional de Saúde e todos os participantes assinaram o Termo de Consentimento Livre e Esclarecido.

## Resultados

Sete famílias que participaram deste estudo foram representadas por um de seus membros e uma (F6) teve dois representantes: a mãe e o padrasto. Entre as pessoas que cometeram suicídio, apenas uma era do sexo feminino (F8); a maioria escolheu o enforcamento como meio de pôr fim a vida (F2, F3, F4, F5, F6, F7 e F8), e somente a F1 foi por intoxicação medicamentosa. As pessoas da F2 e F6 que morreram tinham diagnóstico de esquizofrenia; depressão (F4 e F8) e epilepsia (F1); cinco faziam uso de drogas ilícitas, incluindo cocaína e *crack* (F1, F2, F4, F5, F7 e F8) e maconha (F6); três faziam uso abusivo de álcool (F4, F6 e F7).

O tempo transcorrido desde o suicídio até a coleta de dados variou de sete meses (F4 e F6) a quatro anos (F8). Nas demais, foi um ano (F3), dois anos (F2 e F7) e três anos e meio (F1 e F5). Outros dados que caracterizam os participantes estão apresentados na Figura 1.

**Figura 1-:** Características sociodemográficas dos participantes (n* = 9). Rio Grande, RS, Brasil, 2022

**Características sociodemográficas**								
**Código**	**Parentesco com a vítima**	**Idade (anos)**	**Sexo**	**Status conjugal**	**Raça/Cor**	**Religião**	**Ocupação**	**Escolaridade**
**F1**	Mãe	40	F ^†^	Casada	Preta	Testemunha de Jeová	Diarista	Ensino Médio
**F2**	Irmã	30	F ^†^	Casada	Branca	Espírita	Do lar	Ensino Fundamental II
**F3**	Primo	24	M ^‡^	Solteiro	Branca	Católico	Agente Comunitário de Saúde	Ensino Superior Completo
**F4**	Esposa	38	F ^†^	Viúva	Preta	Evangélica	Confeiteira	Ensino Médio
**F5**	Irmã	27	F ^†^	União Estável	Preta	Umbandista	Desempregada	Ensino Fundamental II
**F6-1**	Mãe	66	F ^†^	União Estável	Preta	Católica	Aposentada e Cuidadora	Ensino Médio
**F6-2**	Padrasto	58	M ^‡^	União Estável	Preta	Católico	Aposentado	Ensino Fundamental I
**F7**	Esposa	39	F ^†^	Viúva	Parda	Macumbeira ^§^	Motorista de Aplicativo	Ensino Médio
**F8**	Esposa	44	F ^†^	Viúva	Branca	Espírita	Chapista	Ensino Médio

*n = Participantes; ^†^F = Feminino; ^‡^M = Masculino; ^§^Termo utilizado pela participante

Os resultados deste estudo são apresentados em quatro núcleos temáticos: (1) *O suicídio como um ato que impacta e extrapola a compreensão*; (2) *A culpa partilhada*; (3) *Sentimentos sobrepostos diante da morte por suicídio*; e (4) *Ensinamentos a partir da experiência*. O primeiro retrata o impacto que a constatação do suicídio provocou individualmente nos membros da família e no grupo como um todo. Os núcleos dois e três mostram os sentimentos mobilizados nos familiares, incluindo a dor pela perda, sobreposta com raiva, alívio e culpa, esta última partilhada com outras pessoas e os serviços sociais e de saúde; o quarto núcleo aborda os ensinamentos que a experiência propicia.

O primeiro núcleo temático, *O suicídio como um ato que impacta e extrapola a compreensão,* mostra as reações de pesar, desespero e perplexidade que os familiares tiveram quando se depararam com o suicídio consumado. As oito famílias descreveram a experiência como trágica e referem que não conseguem entender as razões que levaram a pessoa a tomar uma decisão tão extrema.

Embora para algumas famílias o suicídio fosse uma possibilidade presente no dia a dia, elas se referem ao momento da constatação como inesperado, uma vez que haviam tomado medidas que consideravam preventivas. Na F1, a mãe retirou do alcance do filho objetos que poderiam ser utilizados para enforcamento (cordas), entretanto, ele se suicidou por intoxicação medicamentosa; na F2, a irmã refere que internou o irmão em um hospital psiquiátrico, onde considerava que ele estaria em segurança. Na F6, a mãe procurava estar sempre atenta às alterações na rotina do filho, que morava no mesmo pátio, mas só descobriu o suicídio dois dias após ter sido consumado.

Os depoimentos a seguir mostram o impacto da descoberta do suicídio: *Por ter sido pelo suicídio acho que foi um baque, porque ninguém está preparado para ter um episódio desses na família* (F3); Eu *abri a porta e ele estava ali: o corpo pendurado. Imagina! Eu entrei em desespero* (F4); *Eu pensava: ele não tem onde colocar uma corda, não tenho nada disso em casa, no quarto dele também não tem... Mas ele já tinha planejado o que fazer. Ele tomou todo o estoque de gardenal dele que eu tinha em casa* (F1); *Todo dia de manhã antes de ir trabalhar, eu ia até a porta dele e olhava, eu via a luz acesa, a porta aberta e eu achava que estava tudo bem... Quando eu via a porta fechada é que eu tinha medo de que ele fosse fazer alguma coisa. Passou dois dias que eu não o vi... mas ele já estava lá pendurado* (F6)*.*

O segundo núcleo temático, *A culpa partilhada*, retrata o sentimento de culpa experimentado diante do suicídio cometido por um de seus entes. Este sentimento é expresso pelo familiar de um modo que atribui a contribuição de cada pessoa para o desfecho, semelhante a um processo de partilha, envolvendo três perspectivas: a culpa do próprio participante; a culpa atribuída às outras pessoas do convívio de quem se suicidou e a culpa computada aos serviços sociais e de saúde, por meio dos quais buscavam ajuda.

Os participantes referem que, de alguma maneira, eles próprios contribuíram para o suicídio do familiar, seja por não terem percebido os sinais de que a pessoa não estava bem, seja por não terem tomado atitudes concretas capazes de evitar a morte, e ainda, por não terem se envolvido mais na vida da pessoa que faleceu e consequentemente, não terem a ajudado de forma efetiva.

A segunda perspectiva atribui a culpa a outras pessoas que, segundo a avaliação do participante, contribuíram significativamente para a decisão de o familiar acabar com sua vida. Esta situação é geralmente caracterizada pelo distanciamento físico e emocional de pessoas significativas como pai, mãe e companheiros(as), seja porque priorizavam o trabalho, seja porque deliberadamente escolheram reduzir o contato para evitar conflitos principalmente associados ao uso de drogas e violência.

A terceira perspectiva envolve os serviços sociais e de saúde aos quais a pessoa que se suicidou estava ligada. As oito pessoas que cometeram suicídio eram dependentes de drogas ilícitas ou tinham diagnóstico de transtorno mental e, de alguma forma, estavam vinculadas ao Centro de Atenção Psicossocial Álcool e outras Drogas (CAPS AD) local, ou aos serviços de segurança pública. Entretanto, a morosidade do atendimento nesses serviços fez com que a família só conseguisse o encaminhamento para internação depois do suicídio consumado. *Eu fiz [o registro de ocorrência] só que não adiantou! Tanto que agora é que estão chegando os papeis dele para internar... Agora querem audiência! Vão fazer audiência sem ele aqui? Eu acho que se eles tivessem feito alguma coisa, ele estaria aqui até hoje* (F5). *Ele [a pessoa que se suicidou] passou a noite toda indo na casa do irmão pedir dinheiro e na última vez que ele foi pedir, negou o dinheiro. Não deu meia hora depois a gente ligou avisando que ele estava morto. O irmão se sentiu culpado por não ter dado e isso ele carrega com ele até hoje* (F5).

O sentimento de culpa foi referido pelas oito famílias e sua intensidade variou ao longo do tempo, influenciado pela história vivida anteriormente com a pessoa que morreu; a maneira como os familiares processam a dor; suas crenças e valores.

O terceiro núcleo temático, denominado: *Sentimentos sobrepostos diante da morte por suicídio*, refere-se ao sentimento de dor pela perda, ao qual se somam outros, como o de fracasso na tarefa de cuidar e proteger, mais acentuado quando o vínculo de parentesco era pai/mãe e filho, ou irmãos. Inclui, também, raiva e desejo de vingança, direcionados a outras pessoas que acreditam ter contribuído de forma negativa para o desfecho trágico, como nas relações que envolvem usuários de drogas e traficantes. Inclui, ainda, o sentimento de alívio que experimentam porque, com a morte, encerram um ciclo de relações violentas. Esses sentimentos se manifestam de forma concomitante com a dor pela perda e são influenciados pelos vínculos que mantinham com a pessoa que morreu; com a história vivida com o falecido e com o tempo transcorrido desde a morte por suicídio, como mostram os fragmentos de fala a seguir: *Ele [o pai] entende que falhou, mas é como ele diz: ele viajava! Mas ele entende que falhou e faltou conversar, porque se tu não conversa com teu filho em casa, ele vai procurar os da rua para conversar* (F1); *No meu caso, ao mesmo tempo, foi dor, mas, também, foi um alívio. Não por eu ter me livrado da pessoa, mas porque havia ameaças, havia perseguição* (F7); *Um dia que foi bom para mim, foi quando mataram o traficante que aliciava meu filho. Esse foi o melhor dia para mim! Porque eu vou chorar, mas a mãe dele vai chorar também. A gente não deve desejar isso para os outros, mas é um momento de revolta* (F1).

Os participantes das famílias F1, F2, F4, F5, F6-1, F6-2 e F8 referem que os primeiros momentos após o ocorrido são de extrema dor, como caracterizado na fala de uma mãe: *A sua dor não vai passar! Isto você vai carregar todos os dias contigo. Você vai acordar de manhã e vai pensar na pessoa e sentir a falta dela* (F1).

O quarto núcleo temático, *Ensinamentos a partir da experiência,* mostra que o suicídio consumado passa a ser um marco, integrado na bagagem de experiências das famílias, com capacidade de promover mudanças na dinâmica e no comportamento de seus integrantes. Dentre os ensinamentos referidos pelos participantes estão a necessidade de priorizar a convivência familiar; realizar ajustes na rotina para amenizar a dor e a saudade; aprender a manejar os próprios sentimentos de dor, culpa, raiva; e preservar e valorizar os aspectos positivos da relação com a pessoa que morreu.

Esses ensinamentos assumem papel relevante no processo de reestruturação familiar, com os sobreviventes sentindo-se mais competentes para prevenir ou reduzir a possibilidade de recorrência de suicídio em outros membros da família. Da mesma forma, auxiliam a família a (re)criar uma nova maneira de se relacionar com a perda e a ausência da pessoa que faleceu, como apontam as falas a seguir: *É aprender com a morte para melhorar* (F1); *A dor não dura para sempre... a gente aprende a viver e se apega em tudo que era bom, porque o que era bom conforta e ajuda a seguir em frente* (F2) *Eu consegui parar de me culpar e de me perguntar: onde eu falhei? o que eu fiz? por que eu fui egoísta? Eu resolvi naquele momento dar um basta. São muitas perguntas que não tem respostas! Já não tem volta! mas eu quero viver, então não vou me anular* (F7); *Eu estou aqui hoje [na entrevista] porque eu gostaria de entender melhor as coisas para ajudar a minha filha. Sabe! ela tentou suicídio cinco vezes* (F8).

## Discussão

O suicídio de uma pessoa próxima, como um filho, um pai, um irmão, ou companheiro(a), provoca na unidade familiar uma gama de reações e sentimentos de dor e desespero, além de outros concomitantes de culpa, raiva e alívio, como foi mostrado nos quatro núcleos que retratam os resultados deste estudo. São sentimentos difíceis de conviver, que persistem por um tempo variável para cada pessoa, até um desfecho de reestruturação.

Embora nem todas as famílias que vivenciam uma perda por suicídio consigam se reestruturar como unidade funcional, aquelas que conseguem fazê-lo demonstram algumas características da resiliência familiar. Dentre essas, a presença de um contexto adverso marcado por dor, sofrimento e desespero^(13)^, como mostra o primeiro núcleo. Principalmente quando constataram o suicídio, as famílias referiram que o desespero se manifestou juntamente com o questionamento acerca do porquê a pessoa colocou fim à sua vida. Entretanto, nossos resultados mostram que, à medida que o tempo passa, esses sentimentos vão assumindo novos contornos e os familiares recriam maneiras de se relacionar com a perda e a ausência.

Um estudo realizado na Suécia com famílias sobreviventes corrobora os presentes resultados, demostrando que, mesmo cientes da condição de saúde mental do familiar querido e do expresso desejo, o desejo de morrer, eles acreditavam que o suicídio não seria consumado. Relataram estado de choque com dificuldade de executar as tarefas que a situação exigia^(16)^.

Notadamente, quando o suicídio ocorre na própria residência, são geralmente os familiares que primeiramente encontram o corpo e, nestes casos, a violência desta revelação traz uma sobrecarga emocional adicional para os sobreviventes^(17-18)^, semelhante ao que foi referido por sete famílias do nosso estudo que passaram pela mesma experiência.

Outro estudo sobre a percepção de crianças com menos de seis anos sobre o suicídio de seus pais mostrou que estas se sentiram confusas não exatamente com o suicídio, mas com as reações emocionais dos adultos diante do ocorrido. Muitas delas relataram dificuldade para compreender o que realmente aconteceu^(19)^.

A constatação do suicídio é, sem dúvida, um momento crítico, dependendo das condições em que a morte ocorreu, o que demanda maior atenção à família, incluindo todos os seus membros, de qualquer idade. Nesta situação, é importante não perder de vista que as interações entre os membros da família, com amigos e com os serviços de saúde podem fornecer o apoio de que necessitam, confirmando ser a resiliência moldada em uma rede de relacionamentos e experiências.

Com relação aos sentimentos de culpa que o suicídio de um familiar gera, é importante destacar que estes, mesmo sendo partilhados, não são excludentes. Sentir-se culpado por acreditar que não desempenhou suficientemente bem seu papel de cuidador(a) não exclui a responsabilização dos serviços pela negligência ou a influência de terceiros. É difícil que uma família não se sinta culpada em relação à morte de seu ente querido^(20-21)^, mesmo quando isto é uma probabilidade reconhecida. Especialmente os pais da pessoa que se suicidou costumam responsabilizar-se pela decisão do filho de acabar a vida^(22)^ e, nestes casos, a partilha da culpa pode, de alguma maneira, aliviar o sentimento de vergonha e fracasso no desempenho de seu papel de proteção. Entretanto, o compartilhamento da culpa não elimina a própria culpa.

Embora partilhar possa trazer alívio, autores recomendam atenção, pois a culpa não deve se prolongar a ponto de comprometer a retomada da rotina familiar^(23)^. Por outro lado, compartilhar os sentimentos em grupos de apoio, com outras pessoas que já passaram pela experiência, também ajuda a compreender melhor os fatores que contribuíram para o suicídio e, consequentemente, no processo de enfrentamento e reorganização familiar. Nesse sentido, o grupo de apoio aos familiares constitui-se em um espaço onde aprendem a administrar a autoculpa, reconhecer suas necessidades e descobrir estratégias para manejar o luto^(24)^. Em síntese, vivenciar, partilhar e compartilhar a culpa, enquanto processam seus próprios sentimentos, pode auxiliar no processo de reorganização familiar, inclusive promovendo mudanças no funcionamento da família^(23)^.

A terceira categoria mostra que a morte por suicídio abre espaço para a emergência de outros sentimentos além da dor pela perda, os quais nem sempre são considerados apropriados do ponto de vista social e cultural, para o momento. Trata-se do sentimento de frustração por não ter sido um pai “suficientemente” bom, capaz de proporcionar ao filho o desejo de viver. Esse sentimento, expresso claramente por F1 e F2, é corroborado por outros autores que se referem a esse sentimento de vergonha que leva os pais a isolarem-se socialmente, podendo desenvolver transtornos na saúde física e mental e chegar até tentativas de suicídio. A literatura refere que este comportamento é mais forte entre familiares de primeiro grau e mais frequente nos pais do que nas mães^(23,25)^.

Outro sentimento sobreposto à dor pela perda é a raiva, mais frequentemente dirigida à pessoa que morreu por ter abandonado os que ficaram; ou direcionada a si por não ter impedido a morte^(20-21)^ ou, ainda, a Deus e outras forças incontroláveis, pela incapacidade de explicar o suicídio^(26)^. Um estudo refere-se à raiva voltada aos profissionais de saúde que cuidavam do falecido^(27)^. Este estudo evidenciou a raiva associada a outras pessoas envolvidas com o uso e o tráfico de drogas, em um cenário em que cobrança de dívidas e ameaças à família foram determinantes para a decisão de colocar fim à vida. Nestas situações, além do sentimento de raiva, manifestava-se um desejo de vingança e culpa adicional por não ter evitado que o filho trilhasse o caminho da dependência.

Já o sentimento de alívio sobreposto ao de dor pela perda manifestou-se nas famílias nas quais o relacionamento com a pessoa que se suicidou era de natureza abusiva. Nestes casos, coerente com a literatura, a morte foi um meio de interromper um ciclo de violência^(28)^. Da mesma forma, o sentimento de alívio surge quando a morte põe um ponto final no sofrimento de pessoas com doenças incapacitantes ou mutilantes^(27)^. E, ainda, quando há preocupação com a segurança e o bem-estar do ente querido, geralmente nas situações que envolvem uso e tráfico de drogas. Nestas situações, com o decorrer do tempo, as famílias encontram um “novo normal” e até mesmo a motivação para ajudar outras pessoas que enfrentam situações semelhantes^(29)^.

O quarto núcleo temático retrata uma etapa mais avançada do processo de enfrentamento vivenciado pelas famílias de nosso estudo. Neste, é possível visualizar com nitidez alguns elementos dos processos que ajudam as famílias a superar a dor pela ausência do falecido, tais como: agregar um novo sentido à perda; revisar e reconstruir as relações intrafamiliares, estabelecendo maior coesão entre seus membros; e reconstruir o estilo parental, especificamente nos casos em que o vínculo de parentesco era de pais e filhos.

Autores referem que a família se reorganiza, frequentemente com revisão dos papéis de cada um de seus membros e das responsabilidades parentais^(29-30)^, o que aporta uma bagagem de experiência e habilidades para melhor cuidar de si e dos outros. Além disso, chamam atenção para o fato de que preservar as boas lembranças da relação com a pessoa que morreu ajuda na ressignificação da experiência vivida e na preservação da dignidade do ente querido^(12)^. Assim, na medida em que o tempo passa, a necessidade de encontrar justificativas para o suicídio ocorrido vai diminuindo e abrindo espaço para outras interações familiares^(30-31)^.

Como contribuição deste estudo, destaca-se que focalizar os processos interacionais que sustentam a reestruturação das famílias que vivenciaram a perda de um familiar por suicídio redireciona a ótica que as estigmatiza como problemáticas ou desestruturadas, para as potencialidades destas famílias para enfrentar a situação, se fortalecer e aprender com a experiência. Contribui, também, para alertar que a morte da pessoa “cadastrada no serviço” não encerra a responsabilidade com o cuidado da família enlutada, o que é relevante se considerarmos os altos índices de suicídios e tentativas de suicídio entre pessoas com histórico familiar de tentativas prévias. Nesta situação, cuidar da família enlutada é uma ação preventiva.

Quanto às limitações, cabe destacar o fato de este estudo ter sido realizado com uma amostra reduzida e ter incluído nas entrevistas apenas representantes das famílias enlutadas, o que limitou a identificação dos processos interacionais mobilizados pelos demais segmentos da família.

## Conclusão

A perda de um familiar por suicídio é uma experiência que pode comprometer a unidade familiar se seus membros não encontrarem apoio para resolver os problemas que a situação faz emergir. Com este estudo, foi constatado que a intervenção junto às famílias sobreviventes pode abreviar o tempo necessário para sua reestruturação e que a Atenção Primária à Saúde é a instância na qual ações de cuidado podem ser implementadas de forma efetiva, tendo em vista a facilidade para as equipes identificarem-nas e acessarem-nas.

Nesse contexto, os enfermeiros e demais profissionais da saúde precisam definir as ações de cuidado a partir de avaliações repetidas da repercussão do suicídio na família como um todo e sobre seus membros individualmente; da identificação das dimensões da vida familiar mais comprometidas; das necessidades individuais e familiares; e da mobilização dos processos interacionais de comunicação e organização, os quais favorecem, entre outras coisas, compartilhar a dor e os sentimentos, muitas vezes, contraditório de culpa, raiva e fracasso, entre os membros da própria família, ou com grupos de apoio; além da reorganização da família na totalidade, reconfigurando o lugar e o papel de cada um de seus integrantes. É importante destacar que o cuidado da família sobrevivente não elimina a necessidade de cuidado individualizado de seus membros, uma vez que as pessoas reagem influenciadas pelos vínculos e as histórias que tinham com a pessoa que faleceu.

## Data Availability

Todos os dados gerados ou analisados durante este estudo estão incluídos neste artigo publicado.

# Translation: Familias supervivientes: la experiencia de perder uno de sus miembros por suicidio*

**DOI:** 10.1590/1518-8345.7676.4613

**Authors:** Elena Bustos Alfaro, Mara Regina Santos da Silva, Kateline Simone Gomes Fonseca, Carl Lacharité, Elga Mirta Furtado Barreto de Carvalho, Ariana Sofia Barradas da Silva

**Affiliations:** 1 Universidad de Costa Rica, Escuela de Enfermería, San Pedro, SJ, Costa Rica; 2 Universidade Federal do Rio Grande, Rio Grande, RS, Brasil; 3 Becaria de la Coordenação de Aperfeiçoamento de Pessoal de Nível Superior (CAPES), Brasil; 4 Université Du Québec à Trois Rivières, Quebec, QC, Canadá; 5 Universidade de Cabo Verde, Enfermagem, Praia, Cabo Verde

**Keywords:** Familia, Suicidio completo, Resiliencia Psicológica, Atención Primaria de Salud, Enfermería de la Familia, Salud de la Familia

## Abstract

identificar los procesos interaccionales que permiten a las familias supervivientes a enfrentar la muerte por suicidio, de uno de sus miembros, reestructurarse como unidad y transformar la experiencia en aprendizaje.

estudio cualitativo, exploratorio, orientado por el concepto de Resiliencia Familiar, en el cual participaron ocho familias, que experimentaron el suicidio de uno de sus miembros. La recolección de datos fue realizada por medio de entrevistas semiestructuradas y los datos fueron sometidos al análisis temático.

son presentados en cuatro núcleos temáticos, muestran las reacciones de pesar, desesperación y perplejidad de los familiares, que no consiguen entender las razones para un acto tan radical y los sentimientos de dolor por la pérdida, superpuestos con rabia, alivio y culpa, este último compartido con otras personas y los servicios sociales y de salud; y también se presentan las enseñanzas que la experiencia propicia

los enfermeros y demás profesionales de la salud pueden ayudar a las familias supervivientes a reestructurarse después del suicidio de uno de sus miembros, con acciones de cuidado planeadas a partir de evaluaciones repetidas de la repercusión del suicidio en la familia como un todo y sobre sus miembros individualmente; también se identifican las dimensiones de la vida familiar más comprometidas, las necesidades individuales y familiares, y los procesos de resiliencia familiar.

## Destacados

**(1)** La experiencia del suicidio en la familia desencadena una gama de sentimientos superpuestos. **(2)** Las enseñanzas ayudan a la familia a (re)crear una forma de relacionarse con la pérdida. **(3)** Los profesionales de la salud deben definir el cuidado a partir de evaluaciones constantes.

## Introducción

El suicidio es una de las principales causas de muerte al rededor del mundo, afectando millares de personas y constituyéndose en una experiencia marcada por estigmas debido a su naturaleza inesperada y violenta^(1)^. Cuando afecta a uno de los miembros de la familia, se presenta como una experiencia única, una vez que cada familia percibe y siente de forma diferente. Para algunas familias representa un dolor inconmensurable, en cuanto para otras puede significar, además del dolor, el final de tiempos difíciles y la perspectiva de eliminar o amenizar problemas que experimentados por largo tiempo.

Se estima que, por lo menos, de cinco a seis personas próximas, generalmente de la familia, son afectadas directamente cuando sucede el suicidio de una persona, siendo este un acto que muchas veces estas no consiguen entender^(2-3)^. Considerando que ocurren aproximadamente 700.000 suicidios por año en el mundo, se calcula que cerca de cuatro millones de personas pasan por esa experiencia, lo que representa un gran contingente de familias expuestas que pueden reaccionar de diversas formas frente a este fenómeno^(1)^.

Contribuye para exacerbar el carácter devastador de esa experiencia el hecho de esas familias, en general, no ser el foco de los cuidados en el sistema de salud, convirtiéndose en una población potencialmente vulnerable para desarrollar problemas de salud física y mental, incluyendo ideas o riesgo de suicidio entre sus integrantes^(4-5)^. Muchas veces la atención está centrada en la persona que cometió el suicidio y después del resultado trágico, los familiares quedan desamparados por los servicios de salud, justamente cuando se encuentran en un período de mayor fragilidad, con sentimientos de culpa y rabia exacerbadas^(6-7)^.

Por esa razón, es imprescindible que los enfermeros y otros profesionales, especialmente de la Estrategia Salud de la Familia (ESF), se ocupen de esas familias para ayudarlas en el enfrentamiento de esa experiencia. En ese sentido, es importante que identifiquen la forma cómo cada una reacciona delante de una pérdida y, especialmente, las repercusiones que el suicidio provoca en la familia superviviente, así como las necesidades que presentan. Es en esta perspectiva que la ESF tiene un papel fundamental en el proceso de luto y restructuración de esas familias, justamente porque actúa en una base territorial y comunitaria que facilita el acceso y posibilita conocer las necesidades de cada una^(4)^.

Buscando en las publicaciones nacionales sobre el tema, constatamos un aumento en las tasas de suicidio, en todas las regiones brasileñas, alcanzando 7,27 por cada 100 mil habitantes en 2022, con la región sur presentando el mayor índice 11,53 por cada 100 mil habitantes^(8-9)^. En lo que se refiere a la familia, los estudios enfatizan el impacto del suicidio en los supervivientes de luto incluyendo: el surgimiento o exacerbación de conflictos que pueden llevar a rupturas conyugales y afectivas; el compromiso de los recursos financieros, principalmente si el papel que la persona fallecida ocupaba en la familia era de proveedor; además de trastornos como ansiedad, depresión, abuso de sustancias lícitas e ilícitas, entre otros. Se constata, por otro lado, un vacío en términos de subvención para orientar la práctica de los profesionales^(8-10)^ con estas familias de luto, especialmente en relación a las acciones y estrategias de cuidado, o sea, es un vacío importante en términos de postvención^(4)^.

En la literatura internacional, a pesar de encontrarse en mayor detalle las necesidades de las familias y de recursos de apoyo disponibles principalmente en países desarrollados como Estados Unidos, Australia y Canadá, en donde existen políticas y programas consolidados, los autores refieren que las acciones generalmente son de corto plazo y los cuidados precisan ser calificados, en el sentido de tornarlos resolutivos, sensibles, flexibles y de largo plazo^(5,11-12)^.

Con base en esas consideraciones, orientamos este estudio para los procesos que sustentan la reestructuración de la unidad familiar subsecuente al suicidio de uno de sus miembros, sin perder de vista que esta reestructuración depende del tipo de soporte que las familias encuentran desde el momento en que constatan el suicidio hasta que consigan recuperarse. Algunas familias pueden no disponer de los recursos necesarios para enfrentar esta situación adversa de forma efectiva. En otras, entre tanto, el suicidio de uno de sus miembros desencadena no solo un proceso de luto, pero también de reorganización y reestructuración como unidad familiar, permitiendo que sus integrantes acumulen una variedad de experiencias para administrar otras adversidades, posteriormente^(12-13)^.

Esta situación nos lleva a la concepción de resiliencia familiar, la cual posibilita comprender la forma cómo la familia enfrenta adversidades que impactan su funcionamiento, como la pérdida de un familiar por suicidio y reestructurarse como unidad funcional, apoyada en procesos de interacción de comunicación, organización y de su propio sistema de creencias^(13)^. Los procesos organizacionales contienen, entre otros elementos, la flexibilidad para adaptarse a nuevas situaciones y la conexión entre sus miembros; mientras que los procesos de comunicación están vinculados a la expresión emocional abierta, con claridad y cooperación en la resolución de los problemas; el sistema de creencias está vinculado a la estructura de creencias culturales y religiosas de la familia, que les permite extraer significados de la adversidad, con una perspectiva de confianza y esperanza^(13)^. Se trata de un concepto basal para comprender mejor como las familias se (re)organizan a partir de experiencias adversas que experimentan y la transforman en aprendizaje. En ese sentido, tienen potencial para auxiliar a la intervención en esas familias, objetivando ayudarlas a reestructurarse después del evento traumático.

El objetivo de este estudio es identificar los procesos interaccionales que permiten a las familias supervivientes enfrentar la muerte por suicidio de uno de sus miembros, reestructurarse como unidad y transformar la experiencia en aprendizaje.

## Método

### Tipo de estudio

Estudio cualitativo, de tipo exploratorio, que utilizó como referencia teórica el concepto de Resiliencia Familiar de Froma Walsh^(13)^, una vez que este se aplica a estudios con familias que experimentan situaciones adversas, con fuerte impacto en su funcionamiento, pero a pesar del sufrimiento que experimentan consiguen reestructurarse como unidad funcional y agregar experiencia para enfrentar desafíos, en etapas posteriores de su ciclo vital.

### Local del estudio

El estudio fue desarrollado en el estado de Rio Grande, RS, Brasil, en un municipio en donde, de acuerdo con el Sistema de Notificación, se encuentran altas tasas de suicidio, llegando a 11,55 por 100 mil habitantes; las causas más frecuentes son la baja condición económica de las familias, el consumo de drogas ilícitas y los trastornos depresivos y de ansiedad^(10,14)^.

### Período

Los datos fueron recolectados en el período entre mayo y agosto de 2022.

### Población del estudio

Participaron del estudio ocho familias, representadas por un o dos de sus miembros. Inicialmente, para localizarlas fue realizado un levantamiento de las Declaraciones de muerte (DM) por suicidio, en el Sistema de Información sobre Mortalidad del Servicio de Vigilancia Epidemiológica del municipio, registradas entre enero de 2016 y diciembre de 2021 (período en que los datos estaban disponibles para consultar). Este levantamiento indicó que en 123 familias había ocurrido un caso de suicidio, durante ese período.

Datos obtenidos en las DM como nombre, fecha de nacimiento del fallecido y nombre de los padres, posibilitaron localizar algunos parientes próximos y contactarlos a través de las redes sociales (*Facebook* e *Instagram*). En esta etapa fueron contactadas 14 familias, pero apenas tres aceptaron participar del estudio. Posteriormente fue utilizado el Sistema de Gestantes de la Secretaría Municipal de Salud de Rio Grande, RS (G-MUS) y fueron localizados seis familiares que aceptaron la invitación, pero solamente tres comparecieron a las entrevistas. En un tercer intento para ampliar la muestra, el G-MUS fue nuevamente investigado y fueron localizados 11 familiares. De estos, solamente dos concordaron en participar del estudio. Las familias que rechazaron participar se justificaron por el hecho de ya haber pasado mucho tiempo desde el suicidio y no deseaban revivir los sentimientos dolorosos que la pérdida les ocasionó.

La muestra quedó constituida por las informaciones de ocho familias, seleccionadas de forma intencional, teniendo como base los criterios: haber experimentado el suicidio de un familiar en los cinco años anteriores al inicio de la recolección de datos; residir en el municipio en donde el estudio fue realizado; tener 18 años o más para participar de la entrevista. Como criterio de exclusión fue considerado si el familiar se encontraba en una condición emocional que pudiese comprometer la interacción para realización de las entrevistas, o para hablar sobre el asunto. Las ocho familias fueron identificadas con un código formado por la letra “F” seguida de un numeral correspondiente al orden de realización de las entrevistas.

### Recolección de datos

Los datos fueron recolectados a partir de entrevistas semiestructuradas, realizadas privadamente, de forma presencial con siete familias y una con formato *online*. Todas las entrevistas fueron grabadas (en audio) y realizadas por la primera y tercera autoras de este artículo, enfermeras, doctorandas, residentes en la región en donde el estudio fue desarrollado, integrantes de un grupo de investigación que trabaja con familias en situación de vulnerabilidad y ambas con experiencia previa en recolección de datos cualitativos y sin contacto previo con los participantes.

Para guiar las entrevistas se utilizó una guía constituida de cuatro partes. La primera destinada a obtener datos que caracterizan al informante, en relación a edad, sexo, escolaridad y parentesco con la persona que se suicidó. La segunda orientada para caracterizar a la familia en términos de renta y principal proveedor. La tercera parte trataba de cómo el respondiente y los demás miembros de la familia experimentaron la pérdida del familiar; qué representó el suicidio, o todavía representa en la vida de la familia; qué ayudó a la familia a reestructurarse; las modificaciones que sucedieron en lo cotidiano de la familia después del suicidio; y cómo la familia está organizada actualmente. La cuarta parte investigó en donde y/o con quien los familiares buscaron ayuda antes, durante y después de la persona haberse suicidado; los puntos fuertes de la familia; y los aprendizaje que fueron extraídos de la experiencia. Este guion fue previamente comprobado, siendo que no hubo necesidad de hacer alteraciones.

Las entrevistas duraron en media 2 horas y 40 minutos. Con cuatro familias fueron realizados dos encuentros y con las demás, tres encuentros, totalizando 21 horas y 50 minutos de recolección de datos. A cada nuevo encuentro, los participantes eran convidados a validar las informaciones sintetizadas. Todos fueron convidados para la presentación pública de los resultados, siendo que una participante (F2) asistió a esa ocasión.

### Análisis de los datos

Los datos fueron sometidos al análisis temático^(15)^, siguiendo las etapas: preanálisis, exploración del material y tratamiento de los resultados. Inicialmente, fueron realizadas lecturas sucesivas de todo el material generado en las entrevistas, tratando de identificar patrones y expresiones significativas, que se asociaban a la experiencia del suicidio y elementos de los procesos de resiliencia familiar como cohesión, flexibilidad, entre otros. En la segunda etapa se trató de aprehender los sentidos integrados en las expresiones identificadas anteriormente y crear agrupamientos. Posteriormente, el material fue reagrupado en cuatro núcleos temáticos, utilizando como criterios una dimensión temporal y el sentido asignado por el participante a la pérdida del familiar por suicidio. Cinco investigadores, en diferentes momentos, participaron del proceso de análisis que fue realizado a partir de discusiones presenciales en el formato *online,* no hubo discordancias.

## Aspectos éticos

El estudio recibió certificación del Comité de Ética en Investigación con Seres Humanos de la Universidad Federal de Rio Grande (Edicto: 5.037.888) y del Núcleo Municipal de Educación en Salud Colectiva de la Secretaría Municipal de Salud, con la decisión 13/2022. Fueron respetados los preceptos éticos de la Resolución No. 510/2016 del Consejo Nacional de Salud y todos los participantes firmaron el Término de Consentimiento Libre y Esclarecido.

## Resultados

Las siete familias que participaron de este estudio fueron representadas por uno de sus miembros; una (F6) tubo dos representantes: la madre y el padrastro. Entre las personas que cometieron suicidio, apenas una era del sexo femenino (F8); la mayoría escogió el ahorcamiento como medio de poner fin a la vida (F2, F3, F4, F5, F6, F7 y F8), y solamente la F1 fue por intoxicación medicamentosa. Las personas de la F2 y F6 que murieron tenían diagnóstico de esquizofrenia, depresión (F4 y F8) y epilepsia (F1); cinco utilizaban drogas ilícitas, incluyendo cocaína y *crack* (F1, F2, F4, F5, F7 y F8) y marihuana (F6); tres hacían uso abusivo de alcohol (F4, F6 y F7).

El tiempo transcurrido desde el suicidio hasta la recolección de datos varió de siete meses (F4 y F6) a cuatro años (F8). En las demás fue un año (F3); dos años (F2 y F7); tres años y medio (F1 y F5). Otros datos que caracterizan a los participantes se presentan en la Figura 1.

**Figura 1-:** Características sociodemográficas de los participantes (n* = 9). Rio Grande, RS, Brasil, 2022

**Características sociodemográficas**								
**Código**	**Parentesco con la víctima**	**Edad (años)**	**Sexo**	**Estado conyugal**	**Raza/Color**	**Religi** ón	**Ocupación**	**Escolaridad**
**F1**	Madre	40	F ^†^	Casada	Negra	Testigo de Jehová	Trabajador por día	Enseñanza Media
**F2**	Hermana	30	F ^†^	Casada	Blanca	Espiritista	Tareas domésticas	Enseñanza Fundamental II
**F3**	Primo	24	M ^‡^	Soltero	Blanca	Católico	Agente Comunitario de Salud	Enseñanza Superior Completa
**F4**	Esposa	38	F ^†^	Viuda	Negra	Evangélica	Confitera	Enseñanza Media
**F5**	Hermana	27	F ^†^	Unión Estable	Negra	Umbandista	Desempleada	Enseñanza Fundamental II
**F6-1**	Madre	66	F ^†^	Unión Estable	Negra	Católica	Jubilada y Cuidadora	Enseñanza Media
**F6-2**	Padrastro	58	M ^‡^	Unión Estable	Negra	Católico	Jubilada	Enseñanza Fundamental I
**F7**	Esposa	39	F ^†^	Viuda	Parda	*Macumbeira* ^§^	Taxista	Enseñanza media
**F8**	Esposa	44	F ^†^	Viuda	Blanca	Espírita	Preparación de comida	Enseñanza media

*n = Participantes; ^†^F = Femenino; ^‡^M = Masculino; ^§^Término utilizado por la participante

Los resultados de este estudio se presentan en cuatro núcleos temáticos: (1) *El suicidio como un acto que impacta y extrapola la comprensión*; (2) *La culpa compartida*; (3) *Sentimientos superpuestos delante de la muerte por suicidio*; y (4) *Enseñanzas a partir de la experiencia*. El primero retrata el impacto que la constatación del suicidio provocó, individualmente, en los miembros de la familia y en el grupo como un todo. Los núcleos dos y tres muestran los sentimientos producidos en los familiares, incluyendo el dolor por la pérdida, superpuesto con rabia, alivio y culpa, esta última compartida con otras personas y con los servicios sociales y de salud; el cuarto núcleo aborda las enseñanzas que la experiencia propicia.

El primer núcleo temático, *El suicidio como un acto que impacta y extrapola la comprensión,* muestra las reacciones de pesar, desesperación y perplejidad, que los familiares tuvieron cuando se encontraron con el suicidio consumado. Las ocho familias describieron la experiencia como trágica y refirieron que no consiguen entender las razones que llevaron a la persona a tomar una decisión extrema.

A pesar de que para algunas familias el suicidio fue una posibilidad presente en el día a día, ellas se refieren al momento de la constatación como inesperado, una vez que habían tomado medidas que consideraban preventivas. En la F1, la madre retiró del alcance del hijo objetos que podrían ser utilizados para ahorcarse (cuerdas), entre tanto, él se suicidó por intoxicación medicamentosa; en la F2 la hermana relató que internó al hermano en un hospital psiquiátrico, en el cual consideraba que él estaría en seguridad. En la F6, la madre trataba de estar siempre atenta a las alteraciones en la rutina del hijo, que vivía en el mismo patio, pero solo descubrió el suicidio dos días después de haber sido consumado.

Las declaraciones a seguir muestran el impacto causando por la descubierta del suicidio: *Por haber sido por el suicidio creo que fue un choque, porque nadie está preparado para tener un episodio de esos en la familia* (F3); *Yo abr*í *la puerta y él estaba allí: el cuerpo colgado. ¡Imagina! Yo entré en desesperación* (F4); *Yo pensaba: él no tiene donde colocar una cuerda, no tengo nada de eso en casa, en el cuarto de él también no hay... Pero él ya había planificado que hacer. Él tomó todo la existencia de gardenal que yo tenía en casa* (F1); *Todo día de mañana antes de ir a trabajar, yo iba hasta la puerta de él y miraba, yo veía la luz encendida, la puerta abierta y yo creía que estaba todo bien... Cuando yo veía la puerta cerrada es cuando tenía miedo de que él fuese a hacer alguna cosa. Pasó dos días que yo no lo vi... pero él ya estaba allá colgado* (F6)*.*

El segundo núcleo temático, *La culpa compartida*, retrata el sentimiento de culpa experimentado delante del suicidio cometido por uno de sus miembros. Este sentimiento es expresado por el familiar de un modo que vincula la contribución de cada persona para el desenlace, semejante a un proceso de compartir, envolviendo tres perspectivas: la culpa del propio participante; la culpa atribuida a otras personas que convivieron con quien se suicidó; y, la culpa atribuida a los servicios sociales y de salud, en los cuales buscaban ayuda.

Los participantes refieren que, de alguna manera, ellos propios contribuyeron para el suicidio del familiar, sea por no haber percibido las señales que la persona no estaba bien o por no haber tomado actitudes concretas capaces de evitar la muerte; además, por no haber participado más en la vida de la persona que falleció y consecuentemente no poder ayudar de forma efectiva.

La segunda perspectiva atribuye la culpa a otras personas que, según la evaluación del participante, participaron de forma relevante para la decisión del familiar de acabar con la vida. Esta situación, generalmente es caracterizada por el distanciamiento físico y emocional de personas significativas como padre, madre y compañeros(as), sea porque daban prioridad al trabajo, o porque deliberadamente optaron por reducir el contacto para evitar conflictos principalmente asociados al uso de drogas y violencia.

La tercera perspectiva envuelve a los servicios sociales y de salud a los cuales la persona que se suicidó estaba conectada. Las ocho personas que cometieron el suicidio eran dependientes de drogas ilícitas o tenían diagnóstico de trastorno mental y, de alguna forma, estaban vinculadas al Centro de Atención Psicosocial Alcohol y otras Drogas (CAPS AD) local, o a los servicios de seguridad pública. Entre tanto, la lentitud de la atención en esos servicios permitió que la familia solo consiguiese el encaminamiento para internación después del suicidio consumado. *Yo hice [el registro de ocurrencia] solo que no sirvió. Tanto que unicamente ahora están llegando los papeles de él para internación... ¡Ahora quieren audiencia! ¿Van hacer audiencia sin él aquí? Yo creo que si ellos hubiesen hecho alguna cosa, él estaría aquí hasta hoy* (F5). *Él [la persona que se suicidó] pasó la noche toda yendo a la casa del hermano a pedir dinero y en la última vez que él fue a pedir, negó el dinero. No dio media hora después nos llamaron avisando que él estaba muerto. El hermano se sintió culpado por no haber avisado y él carga con eso hasta hoy* (F5).

El sentimiento de culpa fue referido por las ocho familias y su intensidad varió a lo largo del tiempo, influenciado por: la historia vivida anteriormente con la persona que murió; la manera como los familiares procesan el dolor; y por sus creencias y valores.

El tercer núcleo temático denominado: *Sentimientos superpuestos delante de la muerte por suicidio* se refiere al sentimiento de dolor por la pérdida, a lo que se suman otros como el de fracaso en la tarea de cuidar y proteger, que es más acentuado cuando el vínculo de parentesco era padre/madre e hijo, o hermanos. Incluye también la rabia y deseo de venganza, dirigidos a otras personas que creen que contribuyeron de forma negativa para el desenlace trágico, como en las relaciones que envuelven usuarios de drogas y traficantes. Además, incluye el sentimiento de alivio que experimentan porque con la muerte se encierra un ciclo de relaciones violentas. Esos sentimientos se manifiestan de forma concomitante con el dolor por la pérdida y son influenciados por: los vínculos que mantenían con la persona que murió; con la historia vivida con el fallecido y con el tiempo transcurrido desde la muerte por suicidio, como muestran los fragmentos de declaraciones a seguir: *Él [el padre] entiende que falló, pero es como él dice: él viajaba. Pero él entiende que falló y faltó conversar, porque si tú no conversas con tu hijo en casa, él va buscar a los de la calle para conversar* (F1); *En mí caso, al mismo tiempo, fue dolor, pero, también, fue un alivio. No por haberme librado de la persona, pero porque había amenazas, había persecución* (F7); *un día que fue bueno para mi, fue cuando mataron al traficante que seducía a mí hijo. Ese fue el mejor día para mí. Porque yo voy a llorar, pero la madre de él va llorar también. No debemos desear eso para los otros, pero es un momento de revuelta* (F1).

Los participantes de las familias F1, F2, F4, F5, F6-1, F6-2 y F8 refieren que los primeros momentos después de lo ocurrido son de dolor intenso, como caracterizado en la declaración de una madre: *Tu dolor no va a pasar. Esto usted va a cargar todos los días con usted. Usted va a despertar de mañana y va a pensar en la persona y sentir la falta de ella* (F1).

El cuarto núcleo temático, *Enseñanzas a partir de la experiencia,* muestran que el suicidio consumado pasa a ser un marco, integrado en las experiencias pasadas de las familias, con capacidad de promover cambios en la dinámica y en el comportamiento de sus integrantes. Entre las enseñanzas referidas por los participantes están la necesidades de priorizar la convivencia familiar; realizar ajustes en la rutina para amenizar el dolor y la nostalgia; aprender a manejar los propios sentimientos de dolor, culpa, rabia; y, preservar y valorizar los aspectos positivos de la relación con la persona que murió.

Esas enseñanzas asumen un papel relevante en el proceso de reestructuración familiar, con los supervivientes que se sienten más competentes para prevenir o reducir la posibilidad de recurrencia de suicidio en otros miembros de la familia. De la misma forma, ayudan a la familia a (re)crear una nueva manera de relacionarse con la pérdida y ausencia de la persona que murió, como apuntan las declaraciones a seguir: *Es aprender con la muerte para mejorar* (F1); *El dolor no dura para siempre... necesitamos aprende a vivir y apegarnos a todo lo que era bueno, porque lo que era bueno conforta y ayuda a seguir hacia adelante* (F2) *Yo conseguí parar de culparme y de preguntarme: ¿En donde yo fallé? ¿Qué hice yo? ¿Porqué yo fui egoísta? Yo resolví en aquel momento dar un basta. ¡Son muchas preguntas que no tienen respuestas! ¡Ahora no hay caso! pero yo quiero vivir, entonces no voy a anularme* (F7); *Yo estoy aquí hoy [en la entrevista] porque me gustaría entender mejor las cosas para ayudarme. ¡Sabe! ella trato de suicidarse cinco veces* (F8).

## Discusión

El suicidio de una persona próxima como un hijo, un padre, un hermano, o compañero(a) provoca en la unidad familiar una gama de reacciones y sentimientos de dolor y desesperación, demás de otros sentimientos concomitantes de culpa, rabia y alivio, como fue mostrado en los cuatro núcleos que retratan los resultados de este estudio. Son sentimientos difíciles de convivir, que persisten por un tiempo variable para cada persona, hasta un desenlace de reestructuración.

A pesar de que no todas las familias que experimentan una pérdida por suicidio consiguen reestructurarse como una unidad funcional, aquellas que lo consiguen muestran algunas características de la resiliencia familiar. Entre esas, la presencia de un contexto adverso demarcado por dolor, sufrimiento y desesperación^(13)^, como muestra el primer núcleo. Principalmente cuando constataron el suicidio, las familias refirieron que la desesperación se manifestó juntamente con el cuestionamiento acerca del porque la persona colocó fin a su vida. Entre tanto, nuestros resultados muestran que a medida que el tiempo pasa, esos sentimientos van asumiendo nuevos contornos y los familiares recrean maneras de relacionarse con la pérdida y la ausencia.

Un estudio realizado en Suecia con familias supervivientes corrobora los presentes resultados, mostrando que inclusive consientes de la condición de salud mental del ente querido y del claro deseo de morir, ellos creían que el suicidio no sería consumado y relataron estado de choque con dificultad para ejecutar las tareas que la situación exigía^(16)^.

Señaladamente, cuando el suicidio ocurre en la propia residencia, generalmente son los familiares que primero encuentran el cuerpo y, en estos casos, la violencia de esta revelación produce una sobrecarga emocional adicional para los supervivientes^(17-18)^, semejante a lo que fue referido por siete familias de nuestro estudio que pasaron por la misma experiencia.

Otro estudio sobre la percepción de niños con menos de seis años sobre el suicidio de sus padres, mostró que estas se sintieron confusas no exactamente por el suicidio, pero con las reacciones emocionales de los adultos delante de lo ocurrido. Muchas de ellas relataron dificultad para comprender que fue lo que realmente sucedió^(19)^.

La constatación del suicidio es, sin duda, un momento crítico, dependiendo de las condiciones en que la muerte ocurrió, lo que demanda mayor atención a la familia, incluyendo todos sus miembros, de cualquier edad. En esta situación, es importante no perder de vista que las interacciones entre los miembros de la familia, con amigos y con los servicios de salud puede ofrecer el apoyo que necesitan, confirmando que la resiliencia es moldada en una red de relacionamientos y experiencias.

Con relación a los sentimientos de culpa que el suicidio de un familiar genera, es importante destacar que este, inclusive siendo compartido, no son excluyentes. Sentirse culpado, sea porque cree que no desempeñó suficientemente bien su papel de cuidar, no excluye la responsabilidad de los servicios por negligencia o la influencia de terceros. Es difícil que una familia no se sienta culpada en relación a la muerte de su familiar^(20-21)^, inclusive cuando esto es una probabilidad reconocida. Especialmente los padres de la persona que se suicidó, generalmente, se sienten responsable por la decisión del hijo de acabar con su vida^(22)^ y, en estos casos, el compartir la culpa puede, de alguna manera, aliviar el sentimiento de vergüenza y fracaso en el desempeño de su papel de protección. Entre tanto, el compartir la culpa no elimina la propia culpa.

A pesar de que el compartir pueda aliviar, algunos autores recomiendan prestar atención, ya que la culpa no debe prolongarse al punto de comprometer la retomada de la rutina familiar^(23)^. Por otro lado, compartir los sentimientos en grupos de apoyo, con otras personas que ya pasaron por la experiencia, también ayuda a comprender mejor los factores que contribuyeron para el suicidio y, consecuentemente, el proceso de enfrentamiento y reorganización familiar. En ese sentido, el grupo de apoyo a los familiares es un espacio en donde aprenden a administrar la auto culpa, reconocer sus necesidades y descubrir estrategias para manejar el luto^(24)^. En síntesis, experimentar, compartir y la culpa, en cuanto procesan sus propios sentimientos, puede ayudar en el proceso de reorganización familiar, inclusive promoviendo cambios en el funcionamiento de la familia^(23)^.

La tercera categoría muestra que la muerte por suicidio abre espacio para el surgimiento de otros sentimientos demás del dolor por la pérdida, los cuales no siempre son considerados apropiados, desde el punto de vista social y cultural, para ese momento. Se trata del sentimiento de frustración por no haber sido un padre “suficientemente” bueno, capaz de proporcionar al hijo el deseo de vivir. Ese sentimiento, expresado claramente por F1 y F2, es corroborado por otros autores que se refieren a ese sentimiento de vergüenza que lleva a los padres a aislarse socialmente, pudiendo desarrollar trastornos en la salud física y mental y llegar hasta hacer intentos de suicidio. La literatura refiere que este comportamiento es más fuerte entre familiares de primer grado y más frecuente en los padres de que en las madres^(23,25)^.

Otro sentimiento que se superpone al dolor por la pérdida es la rabia, más frecuentemente dirigida a la persona que murió por haber abandonado a los que quedaron; o dirigida a sí mismo por no haber impedido la muerte^(20-21)^ o, también, a Dios y otras fuerzas incontrolables, por la incapacidad de poder explicar el suicidio^(26)^. Otro estudio se refiere a la rabia dirigida a los profesionales de la salud que cuidaban al fallecido^(27)^. Este estudio mostró la rabia asociada a otras personas envueltas con el uso y el tráfico de drogas, en un escenario en que se cobran deudas y surgen amenazas a la familia, que fueron determinantes para la decisión de colocar fin a la vida. En estas situaciones, además del sentimiento de rabia, se manifiesta un deseo de venganza y culpa adicional por no haber evitado que el hijo se transformase en un dependiente.

Por otro lado el sentimiento de alivio superpuesto al del dolor por la pérdida se manifestó en las familias en la cuales el relacionamiento con la persona que se suicidó era de naturaleza abusiva. En estos casos, coherentemente con la literatura, la muerte fue un medio de interrumpir un ciclo de violencia^(28)^. De la misma forma, el sentimiento de alivio surge cuando la muerte pone punto final al sufrimiento de personas con enfermedades incapacitantes o de mutilaciones^(27)^. También, cuando existe preocupación con la seguridad y el bienestar del ser querido, generalmente en las situaciones que envuelven uso y tráfico de drogas. En estas situaciones, con el transcurso del tiempo, las familias encuentran una “nueva normalidad” e inclusive la motivación para ayudar a otras personas que enfrentan situaciones semejantes^(29)^.

El cuarto núcleo temático retrata una etapa más avanzada del proceso de enfrentamiento experimentado por las familias de nuestro estudio. En este, es posible visualizar con nitidez algunos elementos de los procesos que ayudan a las familias a superar el dolor por la ausencia del fallecido, tales como: agregar un nuevo sentido a la pérdida; revisar y reconstruir las relaciones intrafamiliares, estableciendo mayor cohesión entre sus miembros; y reconstruir el estilo parental, específicamente en los casos en que el vínculo de parentesco era de padres e hijos.

Algunos autores refieren que la familia se reorganiza, frecuentemente, con la revisión de los papeles de cada uno de sus miembros y de las responsabilidades parentales^(29-30)^, lo que aporta experiencias y habilidades para cuidar mejor de sí y de otros. Además de eso, llaman la atención para el hecho de que preservar los buenos recuerdos de la relación con la persona que murió, ayuda en la resignificación de la experiencia vivida y en la preservación de la dignidad del ser querido^(12)^. Así, a medida en que el tiempo pasa, la necesidades de encontrar justificaciones para el suicidio ocurrido va disminuyendo y abriendo espacio para otras interacciones familiares^(30-31)^.

Como contribución de este estudio se destaca que enfocarse en los procesos interaccionales que sustentan la reestructuración de las familias que experimentaron la pérdida de un familiar por suicidio, da una nueva orientación a la perspectiva que las estigmatiza, como problemáticas o desestructuradas, para mostrar las potencialidades de estas familias para enfrentar la situación, se fortalecer y aprender con la experiencia. También, contribuye para alertar que la muerte de la persona “registrada en el servicio” no encierra la responsabilidad con el cuidado de la familia enlutada, lo que es relevante si consideramos los altos índices de suicidios e intentos de suicidio entre personas con histórico familiar de intentos previos. En esta situación, cuidar de la familia enlutada es una acción preventiva.

En cuanto a las limitaciones, cabe destacar que este estudio fue realizado con una muestra reducida y que se incluyeron en las entrevistas apenas con representantes de las familias enlutadas, lo que limitó la identificación de los procesos interaccionales movilizados por los demás segmentos de la familia.

## Conclusión

La pérdida de un familiar por suicidio es una experiencia que puede comprometer la unidad familiar si sus miembros no encuentran apoyo para resolver los problemas que la situación posibilita su surgimiento. En este estudio fue constatado que la intervención junto a las familias supervivientes puede abreviar el tiempo necesario para su reestructuración y que la Atención Primaria de la Salud es la instancia en la cual acciones de cuidado pueden ser implementadas de forma efectiva, considerando la facilidad de los equipos de identificar y acceder a ellas.

En ese contexto, los enfermeros y demás profesionales de la salud precisan: definir las acciones de cuidado a partir de evaluaciones repetidas de la repercusión del suicidio en la familia como un todo y sobre sus miembros individualmente; identificar las dimensiones de la vida familiar más comprometidas y las necesidades individuales y familiares; y, la movilización de los procesos interaccionales de comunicación y organización, los cuales favorecen, entre otras cosas, compartir el dolor y los sentimientos - muchas veces, contradictorios de culpa, rabia y fracaso - entre los miembros de la propia familia o con grupos de apoyo. Además deben participar de la reorganización de la familia como un todo, reconfigurando el lugar y el papel de cada uno de sus integrantes. Es importante destacar que el cuidado de la familia superviviente no elimina la necesidad del cuidado individualizado de sus miembros, ya que las personas reaccionan influenciadas por los vínculos y las historias que tenían con la persona que murió.

## Data Availability

Todos los datos generados o analizados durante este estudio están incluidos en este artículo publicado.
